# HIV-Associated Cryptococcal Meningitis: Bridging the Gap Between Developed and Resource-Limited Settings

**DOI:** 10.1007/s40588-016-0035-5

**Published:** 2016-03-17

**Authors:** Mark W. Tenforde, Rae Wake, Tshepo Leeme, Joseph N. Jarvis

**Affiliations:** Division of Allergy and Infectious Diseases, University of Washington School of Medicine, Seattle, WA USA; University of Washington Medical Center, 1959 NE Pacific Street, Health Sciences Division #356423, Seattle, WA 98195 USA; Institute of Infection and Immunity, St. George’s University of London, London, UK; Centre for Opportunistic, Tropical and Hospital Infections, National Institute for Communicable Diseases, Johannesburg, South Africa; National Institute for Communicable Diseases, 1 Modderfontein Road, Sandringham, Johannesburg, 2131 South Africa; Botswana-UPenn Partnership, Gaborone, Botswana; Division of Infectious Diseases, Department of Medicine, Perelman School of Medicine, University of Pennsylvania, Philadelphia, PA USA; Department of Clinical Research, Faculty of Infectious Diseases and Tropical Medicine, London School of Hygiene and Tropical Medicine, London, UK

**Keywords:** Cryptococcal meningitis, Prevention, Health system strengthening, Resource-limited settings

## Abstract

Cryptococcal meningitis is a major cause of HIV-associated morbidity and mortality worldwide. Most cases occur in low-income countries, where over half of patients die within 10 weeks of diagnosis compared to as few as 10 % of patients from developed countries. A host of factors, spanning the HIV care continuum, are responsible for this gap in treatment outcomes between developed and resource-limited settings. We explore factors responsible for this outcomes gap and describe low-cost, highly effective measures that can be implemented immediately to improve outcomes in resource-limited settings. We also explore health-system challenges that must be addressed to reduce mortality further, recent research in disease prevention, and novel short-course treatment regimens that, if efficacious, could be implemented in resource-limited settings where the cost of standard treatment regimens is currently prohibitive.

## Introduction

Cryptococcal meningitis kills hundreds of thousands of people annually, primarily HIV-infected individuals living in low- and middle-income countries, causing up to 20 % of deaths in HIV-infected cohorts from sub-Saharan Africa (SSA) [1•, 2, 3]. Resource-limited, high-HIV-prevalence countries not only have a far higher incidence of cryptococcal meningitis, but treatment outcomes are dramatically worse than in developed countries. The reasons for these discrepancies are multifold and span the continuum of HIV care: Lack of early HIV diagnosis and initiation of antiretroviral therapy (ART); low retention in care to prevent cryptococcal disease and provide follow-up care after cryptococcal meningitis; poor access to and utilization of sensitive, rapid diagnostics; inadequate access to effective amphotericin-based treatment regimens; and sub-optimal monitoring for and management of drug toxicities, co-morbid conditions, and intracranial pressure (Table 1). Most of these factors arise directly from under-resourcing of health care services in the face of high disease burden. Some simple low-cost, high-impact interventions are implementable immediately to help bridge the outcomes gap between developed and resource-limited settings, while greater health system challenges to effective care will require long-term sustained investment.

**Table 1:** Priorities for improving HIV-associated cryptococcal meningitis outcomes in resource-limited settings

Key recommendation
(1) Early HIV diagnosis and initiation of antiretroviral therapy for primary prevention of cryptococcal meningitis
(2) Serum cryptococcal antigen (CrAg) screening of HIV-infected patients presenting late to care (with CD4 T cell count <100 cells/μL) and pre-emptive antifungal therapy in asymptomatic CrAg-positive patients
(3) Improved health facilities access to lumbar puncture supplies and adequate health care worker training
(4) Rapid diagnostic testing for cryptococcal meningitis in meningitis suspects with highly-sensitive point-of-care CrAg lateral flow assay
(5) Adoption of amphotericin-based induction regimens for cryptococcal meningitis
(6) Access to affordable long-acting flucytosine for induction therapy
(7) Fluid and electrolyte replacement and monitoring during amphotericin-based induction therapy
(8) Routine *therapeutic* lumbar puncture during induction therapy
(9) Diagnosis and management of common co-morbidities, e.g., tuberculosis and bacterial sepsis
(10) Appropriate timing of antiretroviral therapy (~5 weeks) after cryptococcal meningitis and patient retention in care after hospital discharge
(11) Use of maintenance fluconazole to prevent disease relapse

### The Burden of Cryptococcal Meningitis in Developed and Resource-Limited Settings

During the early HIV pandemic in the 1980s, cryptococcal meningitis emerged as a major cause of mortality in HIV-infected individuals. In the USA, Western Europe, and Australia, cryptococcal meningitis occurred in 5–10 % of HIV-infected individuals [4, 5]. After the rollout of ART in the 1990s, the burden of HIV-associated disease fell dramatically in high-income countries. In a study capturing hospital data of over half of the US population, Pyrgos et al. found a 53.6 % decline in hospitalizations from HIV-associated cryptococcal meningitis between 1997 and 2009, compared to a steady rate of non-HIV-associated cryptococcal meningitis [6•]. Similar declines were seen in France, where national surveillance data showed a 46 % decline in the incidence of HIV-associated cryptococcal meningitis in the early post-ART era (1997–2001) compared to pre-ART years (1985–1996) [7], and the UK Collaborative HIV Cohort, which showed a 15-fold decrease in the incidence of HIV-associated cryptococcal meningitis from 1996–1997 to 2006–2007 (from 3.0/1000 to 0.2/1000 person-years) [8].

The vast majority of cryptococcal meningitis cases are now seen in resource-limited countries with high HIV prevalence. Global burden estimates of HIV-associated cryptoccoccal meningitis, published in 2009 based on UNAIDS HIV-prevalence estimates and studies published up to 2007, suggested that of an estimated 957,900 annual cases, over 75 % (720,000) occurred in SSA with most of the rest in South and Southeast Asia [1•]. The marked expansion of ART programs in Africa and Asia since publication of these estimates has almost certainly led to reduction in the number of cases [3], but rates of cryptococcal meningitis remain high [3, 9]. Between November 2010 and October 2012, cryptococcal meningitis was diagnosed in 60 % of all HIV-infected, ART-naïve patients who received a lumbar puncture for suspected meningitis at a tertiary care center in Uganda [10], with similar findings published from Botswana [11], Malawi [12, 13•], South Africa [14], Zambia [15], and Zimbabwe [16]. Pooled estimates from studies in SSA settings published between 1987 and 2014 show cryptococcal meningitis as the laboratory-confirmed etiology in over half of all meningitis cases in HIV-infected patients [17].

### Outcomes of Cryptococcal Meningitis in Developed and Resource-Limited Settings

In developed countries, HIV-infected patients treated for cryptococcal meningitis with standard amphotericin-based induction therapy have a mortality rate as low as 10 % at 10 weeks in prospective randomized-controlled trials (RCTs) [18, 19]. Using more representative “usual care” estimates, a retrospective audit of 24,151 patients hospitalized for HIV-associated cryptococcal meningitis in the USA between 1997 and 2009 found an in-hospital mortality rate of 10.5 % (longer-term mortality estimates were not available) [6•].

Death rates in patients treated for cryptococcal meningitis in resource-limited settings are significantly higher than in developed country settings. In their 2009 Global Burden of Disease paper, Park et al. estimated 3-month HIV-associated cryptococcal meningitis mortality at 55–70 % in low- and middle-income settings and 70 % in SSA [1•]. This is attributable, in part, to the use of inferior fluconazole-based induction regimens in many countries. In prospective observational studies and clinical trials, pooled 10-week mortality of HIV-infected patients receiving high-dose fluconazole induction therapy (800–1200 mg daily) in three Malawian studies was 56 % (70/126) and 54 % (31/57) in a prospective Ugandan RCT [20–23] (Table 2). Even with the use of amphotericin-based regimens, case-fatality rates in resource-limited settings are several-fold higher than in developed country settings. Ten-week mortality with amphotericin-based treatment regimens was 33 % (13/39) in Malawi, 29 % (22/75) and 33 % (16/48) in South African settings, 22 % (14/63) in Thailand, 28 % (8/29) in Uganda, and 36 % (107/298) in Vietnam [24–29]. Long-term follow-up beyond 10 weeks shows even higher mortality rates. Twelve-month survival in clinical trial patients who received amphotericin-based therapy was only 59 % (155/263) in urban South Africa and 44 % (80/182) in urban Uganda [30, 31]. Of note, most of the data above relates to clinical trials, which select for less sick patients and provide unrepresentative care, therefore underestimating mortality in routine care settings. Retrospective reviews of patients treated for HIV-associated cryptococcal meningitis in usual care settings found only 48 % (36/75) of patients survived until discharge at a referral center in Cameroon and 52 % (40/77) at a referral center in Ethiopia [32, 33]. An observational cohort study at a tertiary care center in Uganda found only 41 % (18/44) of patients alive and receiving ART at 6 months after cryptococcal meningitis, and a programmatic review of a district hospital in northern Kwazulu-Natal, South Africa, found only 11 % (8/74) of patients alive and receiving ART at 2 years [34, 35].

**Table 2:** Recent studies with early mortality estimates for cryptococcal meningitis in resource-limited settings

Country	Induction regimen^a^	Mortality, % (n/N)	Clinical trial	Notes	Year	Ref
Botswana	AmB 0.7 mg/kg daily	26 % (7/27) at 24 weeks	Yes	Lower mortality in group that delayed ART for 28 days post-randomization (15 % [2/13]) versus group that initiated ART within 7 days of randomization (36 % [5/14])^b^	2013	[24]
Cameroon	Regimen not specified	52 % (39/75) died in hospital	No		2013	[25]
Ethiopia	Mixture of AmB (*n* = 45) and FLU (*n* = 33)	48 % (37/77) died in hospital	No		2012	[26]
Malawi	FLU 1200 mg daily	55 % (26/47) at 10 weeks	Yes		2014	[21]
Malawi	FLU 800 mg daily	58 % (35/60) at 10 weeks	Yes		2013	[20]
Malawi	AmB 1 mg/kg daily for 7 days + FLU 1200 mg daily; AmB 1 mg/kg daily for 7 days + 5FC 100 mg/kg daily + FLU 1200 mg daily	33 % (13/39) at 10 weeks^b^	Yes	Greater early fungicidal activity with 3-drug regimen	2012	[27]
Malawi	FLU 1200 mg daily; FLU 1200 mg daily + 5FC 100 mg/kg daily	50 % (20/40) at 10 weeks^b^	Yes	Greater early fungicidal activity in combination regimen	2010	[23]
South Africa	Pooled patient population from clinical trials using AmB-based regimen	41 % (108/263) at 1 year	Yes		2014	[28]
South Africa and Uganda	AmB 0.7–1 mg/kg daily + FLU 800 mg daily	38 % (67/177) at 26 weeks	Yes	Lower mortality in group that delayed ART for 5 weeks (30 % [27/89]) versus group that started ART 1–2 weeks after diagnosis (45 % [40/88])	2014	(43)
South Africa	AmB 0.7–1 mg/kg daily +5FC 100 mg/kg daily; AmB 0.7–1 mg/kg daily + FLU 800 mg daily; AmB 0.7–1 mg/kg daily + FLU 600 mg daily; AmB 0.7–1 mg/kg daily + voriconazole 300 mg twice daily	29 % (22/75) at 10 weeks^b^	Yes	No difference in early fungicidal activity between treatment groups	2012	[29]
South Africa	Regimen not specified	89 % (66/74) at 2 years	No		2011	[30]
South Africa	AmB 0.7 mg/kg daily + 5FC 100 mg/kg daily; AmB 1 mg/kg daily + 5FC 100 mg/kg daily	24 % (15/63) at 10 weeks^b^	Yes	Greater early fungicidal activity in group receiving higher dose of AmB	2008	[31]
South Africa	AmB 1 mg/kg daily for 7 days followed by FLU 400 mg daily	33 % (16/48) at 10 weeks	No		2007	[32]
Thailand	AmB 0.7 mg/kg daily; AmB 0.7 mg/kg daily + 5FC 100 mg/kg daily; AmB 0.7 mg/kg daily + FLU 400 mg daily; AmB 0.7 mg/kg daily + 5FC 100 mg/kg daily + FLU 400 mg daily	22 % (14/63) at 10 weeks^b^	Yes	Greater early fungicidal activity with combination AmB + 5FC than other groups	2004	[33]
Uganda	AmB 1 mg/kg daily for 5 days + FLU 1200 mg daily	28 % (8/29) at 10 weeks	Yes		2012	[34]
Uganda	AmB 0.7–1 mg/kg daily	54 % (102/182) at 1 year	No		2012	[35]
Uganda	FLU 800 mg daily; FLU 1200 mg daily	54 % (31/57) at 10 weeks^b^	Yes	Greater early fungicidal activity in group receiving higher dose of FLU	2008	[22]
Uganda	AmB 0.7 mg/kg daily	59 % (26/44) at 6 months	No		2008	[36]
Vietnam	AmB 1 mg/kg daily for 4 weeks; AmB 1 mg/kg daily for 2 weeks + 5FC 100 mg/kg daily; AmB 1 mg/kg daily for 2 weeks + fluconazole 400 mg daily	36 % (107/298) at 10 weeks	Yes	Mortality lower in combined AmB + 5FC group (30 % [30/100]) compared to AmB alone (44 % [44/99])	2013	[37]
Zimbabwe	Fluconazole 800 mg daily	73 % (35/48) at 3 years	Yes	Lower mortality in group that delayed ART for 10 weeks after diagnosis (54 % [12/22]) versus group that started ART within 72 h of diagnosis (88 % [23/26])	2010	[38]

*5FC* flucytosine, *AmB* amphotericin B deoxycholate, *ART* antiretroviral therapy, *FLU* fluconazole

^a^Induction therapy for 14 days unless otherwise noted

^b^No statistically significant difference between groups

### Explaining the Outcomes Gap Between Resource-Rich and Resource-Limited Settings

#### CD4 Counts at ARV Initiation in SSA

HIV diagnosis and ART initiation are delayed in many resource-limited settings, particularly in sub-Saharan Africa, increasing the risk of cryptococcal meningitis and other HIV-associated opportunistic infections [36, 37]. Epidemiological studies from low-income countries generally provide support for incremental gains in CD4 count at ART initiation over time but also find significant heterogeneity between countries, and a majority of patients remain late-presenters to care [38–40]. The International epidemiological Databases to Evaluate AIDS (IeDEA) collaborative cohort estimated a median increase in CD4 count at ART initiation from 2002 to 2009 of 83 to 157 cells/μL in women and 79 to 127 cells/μL in men from low-income countries [40]. These data suggest that a large percentage of HIV-infected individuals are at substantial risk for cryptococcal meningitis, which typically occurs with CD4 count <100 cells/μL [41]. In a combined cohort of patients enrolled in clinical studies for HIV-associated cryptococcal meningitis in Thailand, Malawi, South Africa, and Uganda, the median baseline CD4 count was just 24 cells/μL (IQR 10–50 cells/μL) [31], with similar CD4 counts observed in Botswana [42], Uganda [43••], and Vietnam [29]. Very low baseline CD4 count also increases risk for cryptococcal immune reconstitution inflammatory syndrome (C-IRIS) after the initiation of ART, characterized by an overwhelming inflammatory response against viable or non-viable cryptococci and a high mortality rate [44–46]. Thus, the high prevalence of advanced immune suppression at presentation to HIV-treatment programs explains not only differences in incidence of HIV-associated meningitis between developed and resource-limited settings but also some of the differences in disease severity and mortality rates.

For millions of people living with HIV in resource-limited settings, the largest reduction in cryptococcal meningitis incidence and mortality will be achieved through earlier HIV diagnosis, prompt ART initiation, and successful retention in care. A variety of strategies have been explored to improve HIV diagnosis, such as mobile testing and other community-based testing strategies, home-based testing, and use of rapid point-of-care (POC) tests [47, 48]. Strategies to improve initiation of ART and retention in care, such as health system interventions, behavioral interventions, and financial incentives, have a low supporting evidence base, and additional effort is needed to develop and scale up strategies that are feasible, cost-effective, and acceptable in unique local settings [49, 50].

#### Diagnostic Challenges

A key factor for higher mortality rates observed in resource-limited settings is delayed diagnosis and treatment for cryptococcal meningitis [51, 52]. Diagnosis may be delayed by poor access to health care, failure of health providers to consider cryptococcal meningitis in the differential diagnosis and promptly initiate testing, lack of capacity to perform lumbar puncture (LP), and/or limited availability of rapid and sensitive diagnostic tests.

Diagnosis of cryptococcal meningitis relies on direct visualization of encapsulated yeasts using India ink stain, culture, or immunoassays detecting cryptococcal antigen (CrAg) in the cerebrospinal fluid (CSF). These tests can be cost- and labor-intensive, requiring trained laboratory personnel, equipment, and availability of required reagents. This precludes diagnosis in settings without laboratory facilities or causes a delay while patients or samples are transferred to centers where tests are available [53–55].

India ink stain, widely used in resource-limited settings, is cheap and rapid but has poor sensitivity for diagnosing cryptococcal meningitis [56]. Culture is a relatively cheap option, but it also lacks sensitivity and can take several days to result [53]. While previously available CrAg tests using latex agglutination (LA) and enzyme immunoassay (EIA) require expensive reagents, effective cold chains, laboratory infrastructure and expertise, the recently developed lateral flow assay (LFA, IMMY, Norman, OK) meets the World Health Organization (WHO) ASSURED criteria for diagnostics in resource limited settings (affordable, sensitive and specific, user-friendly, rapid and robust, equipment-free, and deliverable to those who need it) [57]. This test has the potential to revolutionize cryptococcal diagnosis if made widely available. The LFA is cheap (retailing for just US $2 at source) and provides results within 10 min, reducing turnaround time and delays to starting antifungal therapy [56, 58–62, 63••]. It can be stored at room temperature and be performed by health workers with no laboratory training, making it an ideal point-of-care test on blood and CSF [64, 65•]. The LFA performs well in blood and CSF samples [56, 58, 59, 62, 66, 67•, 68, 69], with a review of published studies finding a median 100 % sensitivity and 97.7 % specificity in CSF and 100 % sensitivity and 99.5 % specificity in serum samples [70], and has also been validated using finger-prick capillary blood for meningitis diagnosis [65•], although LP is still needed for pressure reduction and, ideally, to confirm the diagnosis of central nervous system disease.

#### Antifungal Treatment

Optimal antifungal regimens for cryptococcal meningitis have been investigated in clinical trials in both developed and low-resource settings. Recommendations rely on a relatively weak evidence base and are tiered depending on the availability of drugs [51]. Rapid fungal clearance from the CSF during the initiation phase of treatment is clearly important for patient survival [31, 71]. Amphotericin B (AmB) is highly fungicidal and recommended as the foundation for first-line treatment during the initial 2 weeks [51, 72]. This is based on early clinical trial data showing superiority of AmB to fungistatic fluconazole, particularly at higher AmB doses [25, 73, 74], and observational and trial data showing an extremely high mortality in patients treated with fluconazole monotherapy, even at very high doses [20–23].

Flucytosine further increases the rate of fungal clearance when used with AmB induction therapy [24, 28, 75]. A randomized trial in Vietnam demonstrated a significant mortality benefit when flucytosine was used in combination with AmB (1 mg/kg daily) compared to AmB alone in 298 HIV-positive patients (hazard ratio [HR] 0.61, 95 % CI 0.39–0.97) [29]. Due to a lack of flucytosine in most resource-limited settings, when AmB is used, it is usually given in combination with fluconazole. Evidence to inform the optimal dosing of fluconazole is lacking with no confirmed mortality benefit, although the addition of fluconazole to AmB has a favorable effect on early fungal clearance [24, 26, 76, 77].

Despite the survival benefit with the use of amphotericin and flucytosine, the cost, availability, and health system requirements for administering these drugs are prohibitive in most settings where cryptococcal meningitis remains a major cause of death [78]. This continues to be the case despite the inclusion of both AmB and flucytosine on to the WHO list of Essential Medicines in 2013 [79]. Taking into account drug, laboratory, and personnel costs as well as hospital supplies, the cost of first-line 14-day induction therapy with AmB and flucytosine was estimated at US $467.48 per patient in 2012 [80••]. This is unaffordable in a majority of settings despite the fact that this estimate was based on an international wholesale price of flucytosine [78]. A combination of high costs, low demand, and concerns regarding toxicity mean that flucytosine remains unregistered across most of Asia and in all countries in Africa [78].

Aside from cost and availability, toxicities associated with amphotericin and flucytosine, along with the requirement for IV administration of amphotericin, may partly explain the ongoing reliance on fluconazole monotherapy for cryptococcal meningitis in resource-limited settings [81]. Lack of resources to manage drug-related complications and problems related to prolonged hospitalization may also contribute to the persistently higher mortality compared to resource-rich settings [24, 26, 29, 74]. Lipid-based amphotericin formulations are less toxic but are currently unaffordable in most low- and middle-income country settings [78]. However, pre-emptive fluid and electrolyte replacement can reduce the risk of nephrotoxicity with amphotericin, an approach recommended in WHO guidelines for the management of cryptococcal meningitis [51, 82•]. A prospective cohort study conducted in Uganda observed an improvement in 14-day survival from 49 to 62 % (*p* = 0.003) with the introduction of universal administration of 1 L of IV normal saline prior to amphotericin [82•]. Concerns regarding bone marrow toxicity with flucytosine have diminished with clinical trials showing that a lower dose of 100 mg/kg daily is safe and effective and without a need to check therapeutic drug levels as long as complete blood counts are monitored [24, 29].

Shorter courses of amphotericin induction therapy or fully oral regimens with high-dose fluconazole and flucytosine may offer alternative solutions to the current tiered recommendations [23, 27, 28]. A recent analysis concluded that an induction course of AmB (1 mg/kg daily) for 5–7 days and fluconazole 1200 mg daily for 14 days was the most cost-effective regimen compared to fluconazole alone, fluconazole, and flucytosine, and currently recommended first-line therapy of AmB and flucytosine. This regimen provided an additional 4.2 quality adjusted life years (QALYs) at an incremental cost of US $15.11/additional QALY. A 14-day course of flucytosine and fluconazole (1200 mg daily) was the next most cost-effective regimen [80••]. An ongoing phase III randomized controlled trial (Advancing Crytococcal meningitis Treatment in Africa, ACTA, ISRCTN45035509) [46] will verify if these simpler regimens could improve the currently unacceptable levels of cryptococcal-associated mortality [83]. The ongoing phase II trial, AMBITION-cm (ISRCTN10248064), is also evaluating the safety and early fungicidal activity of high-dose liposomal amphotericin (AmBisome) at even shorter courses (as little as 1 dose at 10 mg/kg) with fluconazole 1200 mg daily for 14 days [84].

#### Therapeutic Lumbar Punctures

Raised intracranial pressure (ICP) in cryptococcal meningitis is caused by obstruction to CSF reabsorption at the arachnoid granulations, likely secondary to accumulation of cryptococci and shed capsular polysaccharides, and associated with higher fungal burden [85]. Increased baseline ICP and persistently elevated ICP are associated with neurological sequelae and mortality [86, 87]. The introduction of routine ICP measurement on days 3, 7, and 14, and daily therapeutic LPs until ICP was ≤20 cm H_2_O, for all cases of cryptococcal meningitis in a Tanzanian hospital was associated with a 30-day mortality reduction from 76 to 46 % (HR 2.1, 95 % CI 1.1–3.8) [88]. In addition to decreasing intracranial pressure, lumbar puncture reduces the concentration of capsular polysaccharide (CrAg), potentially providing additional benefit through reduction in cryptococcus and/or shed capsular components [89]. Even after adjusting for baseline CSF opening pressure, at least one therapeutic LP after initial diagnostic LP was associated with a 69 % (95 % CI 18–88 %) improvement in survival compared to no LP in a cohort of 248 patients with HIV-associated cryptococcal meningitis in Uganda and South Africa [90••].

Despite this evidence for the benefit of intensive ICP management, it is often poorly adhered to in resource-limited settings due to lack of equipment and trained personnel, and a poor level of acceptance by patients [34, 88, 91]. Routine scheduling of lumbar punctures during the first 2 weeks of treatment regardless of ICP measurements, as will be included in updated WHO guidelines, will simplify management of raised intracranial pressure and hopefully lead to improved implementation in resource-limited settings. And readily available IV tubing can be used to accurately measure and manage intracranial pressure where the lack of manometers impedes the implementation of serial lumbar punctures [88]. However, efforts must still be made to further understand and address the personal or cultural barriers that inhibit patients from consenting to having LPs in order to improve adherence to this important intervention [91].

#### Post-discharge and Long-Term Management

The early post-hospitalization period is a critical time for patients who have received induction therapy for cryptococcal meningitis. About half of deaths within the first 10 weeks of cryptococcal meningitis diagnosis occur within the first 2 weeks and are primarily related to cryptococcal disease [31]; the remaining deaths in the ensuing weeks are often due to other HIV-associated infections and complications as well as cryptococcal immune reconstitution inflammatory syndrome, highlighting the need for close follow-up and high clinical vigilance. After induction therapy for cryptococcal meningitis with an AmB-based regimen, the COAT trial recently demonstrated a significant 26-week mortality benefit of delayed ART at 5 weeks rather than 1–2 weeks after diagnosis (HR for death 1.73 95 % CI 1.06–2.82) [43••]. Appropriate timing of ART in routine care settings will require careful coordination to prevent both death from premature ART initiation and risk of meningitis relapse or other complications with excessive delay in starting ART. In settings that rely on fluconazole-based induction therapy, early ART is clearly harmful although optimal timing is not clearly established [92].

Many patients with cryptococcal meningitis in resource-limited settings never return to health facilities to receive ART or continuation of maintenance fluconazole therapy (recommended for secondary prevention of cryptococcal meningitis) after they are discharged from the hospital. A retrospective case series in KwaZulu-Natal, South Africa, found that only 9/44 (20 %) of patients collected maintenance phase fluconazole and 11 % (95 % CI 8.7–12.8) were known to be alive and receiving ART after 2 years [35]. In a cohort of patients presenting with recurrent cryptococcal meningitis in South Africa, 30/69 (43 %) were not taking secondary prophylaxis and 8 (12 %) had not commenced ART [93•]. Poor adherence to maintenance fluconazole therapy and low levels of ART initiation lead to a high rate of relapse, which urgently needs addressing in resource-limited settings.

### Prevention of Cryptococcal Meningitis Through CrAg Screening

CrAg testing can be performed on blood samples to screen for patients with early asymptomatic disease who are at a risk of cryptococcal meningitis. Cryptococcal antigenemia is strongly predictive of the development of cryptococcal meningitis in patients with late-stage HIV infection enrolling for ART [94–96]. A “screen-and-treat” strategy for preventing cryptococcal meningitis was conditionally recommended by the WHO in 2011 based on modeling showing that pre-emptive antifungal therapy, if effective in the prevention of downstream clinical disease, is a highly cost-effective strategy [51, 97, 98]. The WHO guidelines recommend that HIV-infected adults with CD4 counts <100 cells/μL and CrAg detected in serum or plasma receive a tapered course of fluconazole (800 mg daily for 2 weeks, followed by 400 mg daily for 8 weeks and then 200 mg daily pending immune reconstitution) as long as they have no signs or symptoms of meningitis (in which case they should receive a lumbar puncture to evaluate for meningitis). This approach has been introduced in a number of developing country settings, and prospective evidence now shows it to be efficacious in reducing the incidence and mortality associated with cryptococcal meningitis [99, 100••, 101, 102]. Several large-scale implementation studies, including the Operational Research for Cryptococcal Antigen Screening (ORCAS) trial in Uganda (NCT01535469), are underway to guide implementation of screening in resource-limited settings [103].

### Conclusions—Closing the Outcomes Gap

In contrast to the 1980s and 1990s, clinical trials for cryptococcal meningitis are now almost exclusively conducted in resource-limited settings. These studies improve our understanding of disease and guide management in resource-poor and resource-rich settings alike [72, 104]. It is imperative, therefore, that not only is cryptococcal meningitis research further expanded in resource-limited settings to provide better prevention and treatment strategies, but that findings from these studies are translated into widespread clinical practice to achieve better outcomes in those regions most affected by this disease.

Studies in the past 5 years demonstrate that several relatively cheap and feasible measures can be implemented that would have a sizable impact on cryptococcal meningitis outcomes. These include cryptococcal antigen screening in vulnerable patients initiating ART; earlier cryptococcal diagnosis through adoption of the point-of-care CrAg lateral flow assay, which is highly sensitive and also cheaper than traditional assays; reliable availability of LP supplies and performance of therapeutic LP as a routine part of care; improved access to AmB and flucytosine; and use of routine IV fluid and electrolytes in patients receiving amphotericin-based treatment. These measures require strengthened health systems with steady supply chains, improved training of front-line health workers, and efforts to understand barriers and improve patient acceptance of LP.

Amphotericin is the backbone of effective treatment for cryptococcal meningitis, and efforts must be made to increase availability in all settings, particularly as high-dose fluconazole has now been shown to be associated with unacceptably poor outcomes (>50 % mortality within 10 weeks of diagnosis). Several phase II and phase III trials are currently underway to evaluate the efficacy of shortened courses of amphotericin-based regimens. If shown to be safe and efficacious, such short course induction could significantly reduce in-hospital time and total costs of treatment and facilitate greater adoption of amphotericin-based treatment in resource-limited settings.

Finally, HIV care is moving increasingly toward a test-and-treat approach given strong evidence for improved long-term patient outcomes and the clear value of HIV treatment as prevention (TasP) [105]. Adoption of earlier diagnosis and ART initiation should ultimately make cryptococcal meningitis a rare disease. This will require not only financial buy-in but also significant investment in operational and implementation strategies to reach vulnerable populations, many of whom are already being missed by current ART programs, and maintain lifelong ART uptake for prevention of cryptococcal meningitis and other HIV-associated diseases. Significant efforts will also be needed to improve coordination of care if CrAg screening for prevention of cryptococcal meningitis is going to be more widely adopted and effective, as well as to prevent loss to follow-up in patients who receive hospital care for incident cryptococcal meningitis. Using the tools we already have, it is possible to make considerable gains in closing the gap in both incidence and outcomes between developed and resource-limited settings for this devastating disease.

## References

[1.•] Park BJ, Wannemuehler KA, Marston BJ, Govender N, Pappas PG, Chiller TM (2009). Estimation of the current global burden of cryptococcal meningitis among persons living with HIV/AIDS. AIDS.

[2] Lawn SD, Harries AD, Anglaret X, Myer L, Wood R (2008). Early mortality among adults accessing antiretroviral treatment programmes in sub-Saharan Africa. AIDS.

[3] Special issue: 9th International Conference on Cryptococcus and Cryptococcosis, 15–19 May 2014, Amsterdam, The Netherlands. Scientific programme. Mycoses, 57:1–4. doi:10.1111/myc.12202. 10.1111/myc.12202 24779363

[4] Powderly WG (1993). Cryptococcal meningitis and AIDS. Clin Infect Dis.

[5] Bicanic T, Harrison TS (2004). Cryptococcal meningitis. Br Med Bull.

[6.•] Pyrgos V, Seitz AE, Steiner CA, Prevots DR, Williamson PR (2013). Epidemiology of cryptococcal meningitis in the US: 1997–2009. PLoS One.

[7] Dromer F, Mathoulin-Pélissier S, Fontanet A, Ronin O, Dupont B, Lortholary O (2004). Epidemiology of HIV-associated cryptococcosis in France (1985–2001): comparison of the pre- and post-HAART eras. AIDS.

[8] Garvey L, Winston A, Walsh J, Post F, Porter K, U. K. Collaborative HIV Cohort Study Steering Committee (2011). HIV-associated central nervous system diseases in the recent combination antiretroviral therapy era. Eur J Neurol.

[9] Jarvis JN, Meintjes G, Wood R, Harrison TS (2010). Testing but not treating: missed opportunities and lost lives in the South African antiretroviral therapy programme. AIDS.

[10] Rajasingham R, Rhein J, Klammer K, Musubire A, Nabeta H, Akampurira A (2015). Epidemiology of meningitis in an HIV-infected Ugandan cohort. AmJTrop Med Hyg.

[11] Mullan PC, Steenhoff AP, Draper H, Wedin T, Bafana M, Anabwani G (2011). Etiology of meningitis among patients admitted to a tertiary referral hospital in Botswana. Pediatr Infect Dis J.

[12] Scarborough M, Gordon SB, Whitty CJ, French N, Njalale Y, Chitani A (2007). Corticosteroids for bacterial meningitis in adults in sub-Saharan Africa. N Engl J Med.

[13.•] Wall EC, Everett DB, Mukaka M, Bar-Zeev N, Feasey N, Jahn A (2014). Bacterial meningitis in Malawian adults, adolescents, and children during the era of antiretroviral scale-up and Haemophilus influenzae type b vaccination, 2000–2012. Clin Infect Dis.

[14] Jarvis JN, Meintjes G, Williams A, Brown Y, Crede T, Harrison TS (2010). Adult meningitis in a setting of high HIV and TB prevalence: findings from 4961 suspected cases. BMC Infect Dis.

[15] Siddiqi OK, Atadzhanov M, Birbeck GL, Koralnik IJ (2010). The spectrum of neurological disorders in a Zambian tertiary care hospital. J Neurol Sci.

[16] Hakim JG, Gangaidzo IT, Heyderman RS, Mielke J, Mushangi E, Taziwa A (2000). Impact of HIV infection on meningitis in Harare, Zimbabwe: a prospective study of 406 predominantly adult patients. AIDS.

[17] Veltman JA, Bristow CC, Klausner JD (2014). Meningitis in HIV-positive patients in sub-Saharan Africa: a review. J Int AIDS Soc.

[18] van der Horst CM, Saag MS, Cloud GA, Hamill RJ, Graybill JR, Sobel JD (1997). Treatment of cryptococcal meningitis associated with the acquired immunodeficiency syndrome. National Institute of Allergy and Infectious Diseases Mycoses Study Group and AIDS Clinical Trials Group. N Engl J Med.

[19] Hamill RJ, Sobel JD, El-Sadr W, Johnson PC, Graybill JR, Javaly K (2010). Comparison of 2 doses of liposomal amphotericin B and conventional amphotericin B deoxycholate for treatment of AIDS-associated acute cryptococcal meningitis: a randomized, double-blind clinical trial of efficacy and safety. Clin Infect Dis.

[20] Rothe C, Sloan DJ, Goodson P, Chikafa J, Mukaka M, Denis B (2013). A prospective longitudinal study of the clinical outcomes from cryptococcal meningitis following treatment induction with 800 mg oral fluconazole in Blantyre, Malawi. PloS one.

[21] Gaskell KM, Rothe C, Gnanadurai R, Goodson P, Jassi C, Heyderman RS (2014). A prospective study of mortality from cryptococcal meningitis following treatment induction with 1200 mg oral fluconazole in Blantyre, Malawi. PloS one.

[22] Longley N, Muzoora C, Taseera K, Mwesigye J, Rwebembera J, Chakera A (2008). Dose response effect of high-dose fluconazole for HIV-associated cryptococcal meningitis in southwestern Uganda. Clin Infect Dis.

[23] Nussbaum JC, Jackson A, Namarika D, Phulusa J, Kenala J, Kanyemba C (2010). Combination flucytosine and high-dose fluconazole compared with fluconazole monotherapy for the treatment of cryptococcal meningitis: a randomized trial in Malawi. Clin Infect Dis.

[24] Brouwer AE, Rajanuwong A, Chierakul W, Griffin GE, Larsen RA, White NJ (2004). Combination antifungal therapies for HIV-associated cryptococcal meningitis: a randomised trial. Lancet.

[25] Bicanic T, Meintjes G, Wood R, Hayes M, Rebe K, Bekker LG (2007). Fungal burden, early fungicidal activity, and outcome in cryptococcal meningitis in antiretroviral-naive or antiretroviral-experienced patients treated with amphotericin B or fluconazole. Clin Infect Dis.

[26] Loyse A, Wilson D, Meintjes G, Jarvis JN, Bicanic T, Bishop L (2012). Comparison of the early fungicidal activity of high-dose fluconazole, voriconazole, and flucytosine as second-line drugs given in combination with amphotericin B for the treatment of HIV-associated cryptococcal meningitis. Clin Infect Dis.

[27] Muzoora CK, Kabanda T, Ortu G, Ssentamu J, Hearn P, Mwesigye J (2012). Short course amphotericin B with high dose fluconazole for HIV-associated cryptococcal meningitis. J Infect.

[28] Jackson AT, Nussbaum JC, Phulusa J, Namarika D, Chikasema M, Kanyemba C (2012). A phase II randomized controlled trial adding oral flucytosine to high-dose fluconazole, with short-course amphotericin B, for cryptococcal meningitis. AIDS.

[29] Day JN, Chau TT, Lalloo DG (2013). Combination antifungal therapy for cryptococcal meningitis. N Engl J Med.

[30] Butler EK, Boulware DR, Bohjanen PR, Meya DB (2012). Long term 5-year survival of persons with cryptococcal meningitis or asymptomatic subclinical antigenemia in Uganda. PLoS One.

[31] Jarvis JN, Bicanic T, Loyse A, Namarika D, Jackson A, Nussbaum JC (2014). Determinants of mortality in a combined cohort of 501 patients with HIV-associated Cryptococcal meningitis: implications for improving outcomes. Clin Infect Dis.

[32] Luma HN, Tchaleu BC, Temfack E, Doualla MS, Ndenga DP, Mapoure YN (2013). HIV-associated central nervous system disease in patients admitted at the Douala General Hospital between 2004 and 2009: a retrospective study. AIDS Res Treat.

[33] Berhe T, Melkamu Y, Amare A (2012). The pattern and predictors of mortality of HIV/AIDS patients with neurologic manifestation in Ethiopia: a retrospective study. AIDS Res Ther.

[34] Kambugu A, Meya DB, Rhein J, O'Brien M, Janoff EN, Ronald AR (2008). Outcomes of cryptococcal meningitis in Uganda before and after the availability of highly active antiretroviral therapy. Clin Infect Dis.

[35] Lessells RJ, Mutevedzi PC, Heller T, Newell ML (2011). Poor long-term outcomes for cryptococcal meningitis in rural South Africa. S Afr Med J.

[36] Hogg RS, Yip B, Chan KJ, Wood E, Craib KJ, O'Shaughnessy MV (2001). Rates of disease progression by baseline CD4 cell count and viral load after initiating triple-drug therapy. JAMA.

[37] Sterne JA, May M, Costagliola D, de Wolf F, Phillips AN, Harris R (2009). Timing of initiation of antiretroviral therapy in AIDS-free HIV-1-infected patients: a collaborative analysis of 18 HIV cohort studies. Lancet.

[38] Siedner MJ, Ng CK, Bassett IV, Katz IT, Bangsberg DR, Tsai AC (2015). Trends in CD4 count at presentation to care and treatment initiation in sub-Saharan Africa, 2002–2013: a meta-analysis. Clin Infect Dis.

[39] Ford N, Mills EJ, Egger M (2015). Editorial commentary: immunodeficiency at start of antiretroviral therapy: the persistent problem of late presentation to care. Clin Infect Dis.

[40] Avila D, Althoff KN, Mugglin C, Wools-Kaloustian K, Koller M, Dabis F (2014). Immunodeficiency at the start of combination antiretroviral therapy in low-, middle-, and high-income countries. J Acquir Immune Defic Syndr.

[41] Jarvis JN, Harrison TS (2007). HIV-associated cryptococcal meningitis. AIDS.

[42] Bisson GP, Molefi M, Bellamy S, Thakur R, Steenhoff A, Tamuhla N (2013). Early versus delayed antiretroviral therapy and cerebrospinal fluid fungal clearance in adults with HIV and cryptococcal meningitis. Clin Infect Dis.

[43.••] Boulware DR, Meya DB, Muzoora C, Rolfes MA, Huppler Hullsiek K, Musubire A (2014). Timing of antiretroviral therapy after diagnosis of cryptococcal meningitis. N Engl J Med.

[44] Chang CC, Dorasamy AA, Gosnell BI, Elliott JH, Spelman T, Omarjee S (2013). Clinical and mycological predictors of cryptococcosis-associated immune reconstitution inflammatory syndrome. AIDS.

[45] Longley N, Harrison TS, Jarvis JN (2013). Cryptococcal immune reconstitution inflammatory syndrome. Curr Opin Infect Dis.

[46] Grant PM, Komarow L, Andersen J, Sereti I, Pahwa S, Lederman MM (2010). Risk factor analyses for immune reconstitution inflammatory syndrome in a randomized study of early vs. deferred ART during an opportunistic infection. PloS one.

[47] Sabapathy K, Van den Bergh R, Fidler S, Hayes R, Ford N (2012). Uptake of home-based voluntary HIV testing in sub-Saharan Africa: a systematic review and meta-analysis. PLoS Med.

[48] Suthar AB, Ford N, Bachanas PJ, Wong VJ, Rajan JS, Saltzman AK (2013). Towards universal voluntary HIV testing and counselling: a systematic review and meta-analysis of community-based approaches. PLoS Med.

[49] Nachega JB, Uthman OA, del Rio C, Mugavero MJ, Rees H, Mills EJ (2014). Addressing the Achilles’ heel in the HIV care continuum for the success of a test-and-treat strategy to achieve an AIDS-free generation. Clin Infect Dis.

[50] Govindasamy D, Meghij J, Kebede Negussi E, Clare Baggaley R, Ford N, Kranzer K (2014). Interventions to improve or facilitate linkage to or retention in pre-ART (HIV) care and initiation of ART in low- and middle-income settings—a systematic review. J Int AIDS Soc.

[51] World Health Organization (2011). Rapid advice: diagnosis, prevention and management of cryptococcal disease in HIV-infected adults, adolescents and children.

[52] Trachtenberg JD, Kambugu AD, McKellar M, Semitala F, Mayanja-Kizza H, Samore MH (2007). The medical management of central nervous system infections in Uganda and the potential impact of an algorithm-based approach to improve outcomes. Int J Infect Dis.

[53] Nelson MR, Fisher M, Cartledge J, Rogers T, Gazzard BG (1994). The role of azoles in the treatment and prophylaxis of cryptococcal disease in HIV infection. AIDS.

[54] Petti CA, Polage CR, Quinn TC, Ronald AR, Sande MA (2006). Laboratory medicine in Africa: a barrier to effective health care. Clin Infect Dis.

[55] Mundy C, Ngwira M, Kadewele G, Bates I, Squire SB, Gilks CF (2000). Evaluation of microscope condition in Malawi. Trans R Soc Trop Med Hyg.

[56] Boulware DR, Rolfes MA, Rajasingham R, von Hohenberg M, Qin Z, Taseera K (2014). Multisite validation of cryptococcal antigen lateral flow assay and quantification by laser thermal contrast. Emerg Infect Dis.

[57] Drain PK, Hyle EP, Noubary F, Freedberg KA, Wilson D, Bishai WR (2014). Diagnostic point-of-care tests in resource-limited settings. Lancet Infect Dis.

[58] Hansen J, Slechta ES, Gates-Hollingsworth MA, Neary B, Barker AP, Bauman S (2013). Large-scale evaluation of the immuno-mycologics lateral flow and enzyme-linked immunoassays for detection of cryptococcal antigen in serum and cerebrospinal fluid. Clin Vaccine Immunol.

[59] Lindsley MD, Mekha N, Baggett HC, Surinthong Y, Autthateinchai R, Sawatwong P (2011). Evaluation of a newly developed lateral flow immunoassay for the diagnosis of cryptococcosis. Clin Infect Dis.

[60] Kozel TR, Bauman SK (2012). CrAg lateral flow assay for cryptococcosis. Expert Opin Med Diagn.

[61] Lourens A, Jarvis JN, Meintjes G, Samuel CM (2014). Rapid diagnosis of cryptococcal meningitis by use of lateral flow assay on cerebrospinal fluid samples: influence of the high-dose “hook” effect. J Clin Microbiol.

[62] McMullan BJ, Halliday C, Sorrell TC, Judd D, Sleiman S, Marriott D (2012). Clinical utility of the cryptococcal antigen lateral flow assay in a diagnostic mycology laboratory. PLoS One.

[63.••] Kabanda T, Siedner MJ, Klausner JD, Muzoora C, Boulware DR (2014). Point-of-care diagnosis and prognostication of cryptococcal meningitis with the cryptococcal antigen lateral flow assay on cerebrospinal fluid. Clin Infect Dis.

[64] Shroufi A. Lay cadre point of care CRAG screening in Lesotho. SA AIDS; 2015 June; Durban, South Africa.

[65.•] Williams DA, Kiiza T, Kwizera R, Kiggundu R, Velamakanni S, Meya DB, et al. Evaluation of fingerstick cryptococcal antigen lateral flow assay in hiv-infected persons: a diagnostic accuracy study. Clin Infect Dis. 2015;61(3):464–7. **Study showing that fingerstick (capillary blood) CrAg lateral flow assay testing performs well in screening blood for cryptococcosis, with 100% agreement between fingerstick and plasma/serum blood testing. This method of screening early cryptococcal infection for prevention of cryptococcal meningitis might be ideal in settings with limited access to phlebotomy supplies.** 10.1093/cid/civ263 PMC4503809 25838287

[66] Binnicker MJ, Jespersen DJ, Bestrom JE, Rollins LO (2012). Comparison of four assays for the detection of cryptococcal antigen. Clin Vaccine Immunol.

[67.•] Jarvis JN, Percival A, Bauman S, Pelfrey J, Meintjes G, Williams GN (2011). Evaluation of a novel point-of-care cryptococcal antigen test on serum, plasma, and urine from patients with HIV-associated cryptococcal meningitis. Clin Infect Dis.

[68] Rugemalila J, Maro VP, Kapanda G, Ndaro AJ, Jarvis JN (2013). Cryptococcal antigen prevalence in HIV-infected Tanzanians: a cross-sectional study and evaluation of a point-of-care lateral flow assay. Trop Med Int Health.

[69] Escandon P, Lizarazo J, Agudelo CI, Chiller T, Castaneda E (2013). Evaluation of a rapid lateral flow immunoassay for the detection of cryptococcal antigen for the early diagnosis of cryptococcosis in HIV patients in Colombia. Med Mycol.

[70] Vijayan T, Chiller T, Klausner JD (2013). Sensitivity and specificity of a new cryptococcal antigen lateral flow assay in serum and cerebrospinal fluid. MLO Med Lab Observer.

[71] Bicanic T, Muzoora C, Brouwer AE, Meintjes G, Longley N, Taseera K (2009). Independent association between rate of clearance of infection and clinical outcome of HIV-associated cryptococcal meningitis: analysis of a combined cohort of 262 patients. Clin Infect Dis.

[72] Perfect JR, Dismukes WE, Dromer F, Goldman DL, Graybill JR, Hamill RJ (2010). Clinical practice guidelines for the management of cryptococcal disease: 2010 update by the Infectious Diseases Society of America. Clin Infect Dis.

[73] Saag MS, Powderly WG, Cloud GA, Robinson P, Grieco MH, Sharkey PK (1992). Comparison of amphotericin B with fluconazole in the treatment of acute AIDS-associated cryptococcal meningitis. The NIAID Mycoses Study Group and the AIDS Clinical Trials Group. N Engl J Med.

[74] Bicanic T, Wood R, Meintjes G, Rebe K, Brouwer A, Loyse A (2008). High-dose amphotericin B with flucytosine for the treatment of cryptococcal meningitis in HIV-infected patients: a randomized trial. Clin Infect Dis.

[75] Schwarz P, Dromer F, Lortholary O, Dannaoui E (2006). Efficacy of amphotericin B in combination with flucytosine against flucytosine-susceptible or flucytosine-resistant isolates of Cryptococcus neoformans during disseminated murine cryptococcosis. Antimicrob Agents Chemother.

[76] Larsen RA, Bauer M, Thomas AM, Graybill JR (2004). Amphotericin B and fluconazole, a potent combination therapy for cryptococcal meningitis. Antimicrob Agents Chemother.

[77] Diamond DM, Bauer M, Daniel BE, Leal MA, Johnson D, Williams BK (1998). Amphotericin B colloidal dispersion combined with flucytosine with or without fluconazole for treatment of murine cryptococcal meningitis. Antimicrob Agents Chemother.

[78] Loyse A, Dromer F, Day J, Lortholary O, Harrison TS (2013). Flucytosine and cryptococcosis: time to urgently address the worldwide accessibility of a 50-year-old antifungal. J Antimicrob Chemother.

[79] World Health Organization. 19th WHO model list of essential medicines.2015 Oct 20 2015.

[80.••] Rajasingham R, Rolfes MA, Birkenkamp KE, Meya DB, Boulware DR (2012). Cryptococcal meningitis treatment strategies in resource-limited settings: a cost-effectiveness analysis. PLoS Med.

[81] Bicanic T, Bottomley C, Loyse A, Brouwer AE, Muzoora C, Taseera K (2015). Toxicity of Amphotericin B deoxycholate-based induction therapy in patients with HIV-associated cryptococcal meningitis. Antimicrobial agents and chemotherapy..

[82.•] Bahr NC, Rolfes MA, Musubire A, Nabeta H, Williams DA, Rhein J (2014). Standardized electrolyte supplementation and fluid management improves survival during amphotericin therapy for cryptococcal meningitis in resource-limited settings. Open Forum Infect Dis.

[83] St. George’s University of London. A phase III, randomised, controlled trial for the treatment of HIV-associated cryptococcal meningitis: oral fluconazole plus flucytosine or one week amphotericin B-based therapy vs two weeks amphotericin B-based therapy http://www.controlled-trials.com/ISRCTN45035509?q=&filters=conditionCategory:Infections and Infestations,recruitmentCountry:Cameroon&sort = &offset = 1&totalResults = 8&page = 1&pageSize = 50&searchType = basic-search2012 [cited 2015 Oct 20].

[84] Molefi M, Chofle AA, Molloy SF, Kalluvya S, Changalucha JM, Cainelli F (2015). AMBITION-cm: intermittent high dose Am Bisome on a high dose fluconazole backbone for cryptococcal meningitis induction therapy in sub-Saharan Africa: study protocol for a randomized controlled trial. Trials.

[85] Loyse A, Wainwright H, Jarvis JN, Bicanic T, Rebe K, Meintjes G (2010). Histopathology of the arachnoid granulations and brain in HIV-associated cryptococcal meningitis: correlation with cerebrospinal fluid pressure. AIDS.

[86] Bicanic T, Brouwer AE, Meintjes G, Rebe K, Limmathurotsakul D, Chierakul W (2009). Relationship of cerebrospinal fluid pressure, fungal burden and outcome in patients with cryptococcal meningitis undergoing serial lumbar punctures. AIDS.

[87] Graybill JR, Sobel J, Saag M, van Der Horst C, Powderly W, Cloud G (2000). Diagnosis and management of increased intracranial pressure in patients with AIDS and cryptococcal meningitis. The NIAID Mycoses Study Group and AIDS Cooperative Treatment Groups. Clin Infect Dis.

[88] Meda J, Kalluvya S, Downs JA, Chofle AA, Seni J, Kidenya B (2014). Cryptococcal meningitis management in Tanzania with strict schedule of serial lumber punctures using intravenous tubing sets: an operational research study. J Acquir Immune Defic Syndr.

[89] Wijewardana I, Jarvis JN, Meintjes G, Harrison TS, Bicanic T (2011). Large volume lumbar punctures in cryptococcal meningitis clear cryptococcal antigen as well as lowering pressure. J Infect.

[90.••] Rolfes MA, Hullsiek KH, Rhein J, Nabeta HW, Taseera K, Schutz C (2014). The effect of therapeutic lumbar punctures on acute mortality from cryptococcal meningitis. Clin Infect Dis.

[91] Thakur KT, Mateyo K, Hachaambwa L, Kayamba V, Mallewa M, Mallewa J (2015). Lumbar puncture refusal in sub-Saharan Africa: a call for further understanding and intervention. Neurology.

[92] Makadzange AT, Ndhlovu CE, Takarinda K, Reid M, Kurangwa M, Gona P (2010). Early versus delayed initiation of antiretroviral therapy for concurrent HIV infection and cryptococcal meningitis in sub-saharan Africa. Clin Infect Dis.

[93.•] Jarvis JN, Meintjes G, Williams Z, Rebe K, Harrison TS (2010). Symptomatic relapse of HIV-associated cryptococcal meningitis in South Africa: the role of inadequate secondary prophylaxis. S Afr Med J.

[94] Jarvis JN, Lawn SD, Vogt M, Bangani N, Wood R, Harrison TS (2009). Screening for cryptococcal antigenemia in patients accessing an antiretroviral treatment program in South Africa. Clin Infect Dis.

[95] French N, Gray K, Watera C, Nakiyingi J, Lugada E, Moore M (2002). Cryptococcal infection in a cohort of HIV-1-infected Ugandan adults. AIDS.

[96] Liechty CA, Solberg P, Were W, Ekwaru JP, Ransom RL, Weidle PJ (2007). Asymptomatic serum cryptococcal antigenemia and early mortality during antiretroviral therapy in rural Uganda. Trop Med Int Health.

[97] Jarvis JN, Harrison TS, Lawn SD, Meintjes G, Wood R, Cleary S (2013). Cost effectiveness of cryptococcal antigen screening as a strategy to prevent HIV-associated cryptococcal meningitis in South Africa. PLoS One.

[98] Meya DB, Manabe YC, Castelnuovo B, Cook BA, Elbireer AM, Kambugu A (2010). Cost-effectiveness of serum cryptococcal antigen screening to prevent deaths among HIV-infected persons with a CD4+ cell count < or =100 cells/microL who start HIV therapy in resource-limited settings. Clin Infect Dis.

[99] Pac L, Horwitz MM, Namutebi AM, Auerbach BJ, Semeere A, Namulema T (2015). Implementation and operational research: integrated pre-antiretroviral therapy screening and treatment for tuberculosis and cryptococcal antigenemia. J Acquir Immune Defic Syndr.

[100.••] Mfinanga S, Chanda D, Kivuyo SL, Guinness L, Bottomley C, Simms V (2015). Cryptococcal meningitis screening and community-based early adherence support in people with advanced HIV infection starting antiretroviral therapy in Tanzania and Zambia: an open-label, randomised controlled trial. Lancet.

[101] Meya D, Rajasingham R, Nalintya E, Tenforde M, Jarvis JN (2015). Preventing cryptococcosis-shifting the paradigm in the era of highly active antiretroviral therapy. Curr Trop Med Rep.

[102] Longley N, Jarvis JN, Meintjes G, Boulle A, Cross A, Kelly N (2015). Cryptococcal antigen screening in patients initiating art in South Africa: a prospective cohort study. Clin Infect Dis.

[103] University of Minnesota Clinical and Translational Science Institute. Operational research for cryptococcal antigen screening (ORCAS) https://clinicaltrials.gov/ct2/show/NCT015354692015.

[104] Manabe YC, Moore RD (2015). Cryptococcal antigen screening and preemptive treatment in a US cohort of patients with AIDS. Clin Infect Dis.

[105] Cohen MS, Chen YQ, McCauley M, Gamble T, Hosseinipour MC, Kumarasamy N (2011). Prevention of HIV-1 infection with early antiretroviral therapy. N Engl J Med.

